# Pridopidine Protects ALS Patient-Derived Neural Progenitor Cells via Sigma-1 Receptor Activation

**DOI:** 10.3390/ijms27083489

**Published:** 2026-04-14

**Authors:** May Meltzer, Maya Shefler Zamir, Noam Tzuri, Andrew M. Tan, Michal Geva, Michael R. Hayden, Rachel G. Lichtenstein

**Affiliations:** 1 Prilenia Therapeutics B.V., 1411 DC Naarden, The Netherlands; 2 Avram and Stella Goren-Goldstein Department of Biotechnology Engineering, Faculty of Engineering, Ben-Gurion University of the Negev, P.O. Box 653, Beer-Sheva 84105, Israel; 3 Department of Neurology, Yale University School of Medicine, New Haven, CT 06510, USA; 4 Regenerative Medicine and Stem Cell (RMSC) Research Center, Ben-Gurion University of the Negev, P.O. Box 653, Beer-Sheva 84105, Israel; 5 Department of Medical Genetics, Centre for Molecular Medicine and Therapeutics, University of British Columbia, Vancouver, BC V5Z 4H4, Canada

**Keywords:** pridopidine, amyotrophic lateral sclerosis, Sigma-1 receptor, endoplasmic reticulum stress, mitochondrial membrane potential, mitochondria-associated membranes, iPSC-derived neural progenitor cells

## Abstract

The sigma-1 receptor (S1R) is an endoplasmic reticulum (ER)-resident protein enriched at the mitochondria-associated ER membranes (MAMs) that supports ER homeostasis, preserves mitochondrial function, and enhances cell survival under stress. Disruptions of MAM integrity and prolonged ER stress are well-recognized pathological features of amyotrophic lateral sclerosis (ALS), contributing to motor neuron dysfunction and degeneration. In this study, we evaluated the protective effects of pridopidine, a highly selective and potent S1R agonist currently in clinical development for Huntington’s disease (HD) and ALS, using neural progenitor cells (NPCs) derived from induced pluripotent stem cells (iPSCs) from a patient with sporadic ALS. Exposure of ALS NPCs to the ER stressor tunicamycin increased the ER stress markers binding immunoglobulin protein (BiP) and C/EBP homologous protein (CHOP), disrupted mitochondrial membrane potential, upregulated expression of the mitochondrial apoptotic marker, *BAX*, increased caspase-3 activation, and reduced cell viability. Pridopidine significantly attenuated tunicamycin-induced BiP and CHOP expression in a biphasic, dose-dependent manner (with maximal efficacy at 1 µM), consistent with the typical pharmacology of S1R agonists. Pridopidine restored mitochondrial membrane potential, reduced mitochondrial apoptotic signaling, shown by decreased *BAX* expression and caspase-3 activation, and improved survival of ALS-NPCs under ER stress. Co-treatment with the selective S1R antagonist, NE-100, attenuated these effects, supporting an S1R-mediated mechanism of action for pridopidine. Together, these results demonstrate that S1R activation by pridopidine mitigates ER-stress-induced mitochondrial dysfunction and cell loss in ALS-NPCs, resulting in enhanced survival of NPCs supporting the therapeutic potential of pridopidine in ALS.

## 1. Introduction

ALS is a progressive and fatal neurodegenerative disorder characterized by selective loss of motor neurons, leading to muscle atrophy, paralysis, and respiratory failure, with death often occurring within 3–5 years of symptom onset [1,2,3]. Current treatment options are either limited to a small and selective target population of SOD1 mutation carriers [4] or show limited benefits and do not alter disease progression [5,6]. Thus, there is a critical unmet need for effective disease-modifying therapies [7,8,9,10].

Sporadic and SOD1-mutant ALS types converge on shared cellular pathways that underline motor neuron degeneration. These forms of ALS share key cellular pathologies, including impaired ER–mitochondria crosstalk at the MAMs, prolonged ER stress, formation of toxic protein aggregates, mitochondrial dysfunction, and progressive loss of motor neurons in the spinal cord and cortex [3,11,12,13,14].

Among these processes associated with ALS, ER stress has emerged as one of the earliest pathogenic mechanisms [15,16,17,18]. Increased expression of ER stress markers, including the chaperone BiP/GRP78 and the pro-apoptotic transcription factor CHOP, has been consistently observed in ALS patient tissues and preclinical models [16,19,20,21]. Prolonged ER stress also contributes to neuronal loss in ALS by activating apoptotic signaling, including upregulation of the pro-apoptotic mediator, BAX, and downstream activation of caspase-3 [20,22].

Importantly, the S1R plays a central role in stabilizing MAMs integrity and regulating ER stress responses [19,23,24]. In healthy unstressed cells, the S1R is bound to BiP at the ER membrane in an inactive manner. Upon ER calcium depletion and elevated ER stress, or agonist binding, the S1R dissociates from BiP and interacts with other cellular proteins to reduce stress and enhance cell survival [25,26]. S1R interacts and stabilizes the inositol 1,4,5-trisphosphate receptors (IP3Rs), responsible for Ca^2+^ transfer from ER stores to the mitochondria, normalizing calcium homeostasis, reducing ER stress and enhancing mitochondrial function [27,28,29].

Genetic evidence supports the role of S1R in ALS. For example, complete loss-of-function mutations in *SIGMAR1* cause juvenile ALS and distal hereditary motor neuropathies, while partial loss-of-function variants increase risk for adult-onset ALS. Consistent with these genetic findings, pharmacological S1R activation mitigates ER stress, enhances mitochondrial function, stabilizes calcium homeostasis, and promotes neuronal survival across multiple neurodegeneration models, including ALS, HD, Parkinson’s disease (PD), and Alzheimer’s disease (AD) [3,30,31,32,33,34].

In both in vitro and in vivo preclinical ALS models, pridopidine, via activation of the S1R, restores axonal transport, preserves neuromuscular junction integrity, reduces toxic mutant SOD1 aggregation, and improves motor neuron survival [35,36,37]. Beyond ALS, pridopidine stabilizes dendritic spines and enhances synaptic plasticity in association with improved cognitive or motor outcomes in HD, PD and AD models. These broad neuroprotective actions are consistent with S1R’s role as a key regulator of ER–mitochondria crosstalk, calcium homeostasis, mitochondrial function, and cellular stress responses [37,38,39,40,41].

A well-established fundamental property of S1R agonist activity is the biphasic dose–response curve, extensively demonstrated in preclinical models and in human studies [41,42]. Evidence for this dose–response characteristic of S1R agonists comes from various preclinical assays, including amelioration of cognitive impairment [43,44,45,46,47,48,49,50], electrophysiological measure of N-methyl-d-aspartic acid (NMDA)-induced neuronal activation in rat brains [51,52,53], potentiation of cellular calcium efflux [54,55], and anti-depressive effects in mice [56]. Pridopidine demonstrated a biphasic dose response in several nonclinical studies. Pridopidine restored the impaired homeostatic synaptic plasticity in HD cortical neurons with the strongest effect achieved at a concentration of 1 μM, while higher and lower doses showed diminished effects [57]. In the 6-OHDA PD mouse model, a low dose of pridopidine, but not a high dose, increased neuroprotection of dopaminergic neurons and restored behavioral abnormalities [58].

In this study, we investigated the cellular effects of pridopidine under ER stress conditions using NPCs derived from a sporadic ALS patient. Specifically, we evaluated whether pridopidine reduces ER stress by examining BiP and CHOP expression; restores mitochondrial membrane potential and reduces mitochondrial apoptotic signaling as measured by *BAX* expression and caspase-3 activation; and improves cell viability.

## 2. Results

### 2.1. Differentiation of Sporadic ALS Patient iPSCs into Neural Progenitor Cells

**Figure 1:**
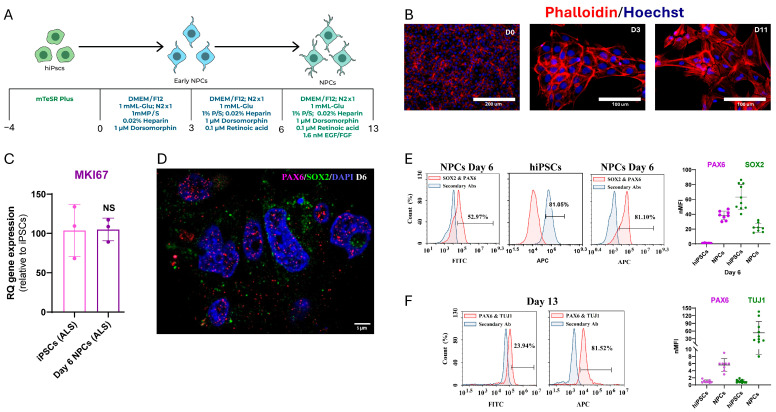
Directed differentiation of sporadic ALS patient-derived iPSCs into NPCs and early neurons. (**A**) Schematic representation of the stepwise neural induction protocol used to differentiate ALS patient- iPSCs into NPCs and neurons. Cells were cultured in mTeSR Plus medium until day 0, then sequentially transitioned through early NPC and NPC media containing DMEM/F12, N2, heparin, dorsomorphin, and retinoic acid, with final maturation in EGF/FGF-containing medium. (**B**) Representative immunofluorescence images show cytoskeletal organization during differentiation at day 0, day 3, and day 11 stained with Phalloidin (red, F-actin) and Hoechst (blue, nuclei). Scale bars represent 100/200 mm (n = 3 biological). (**C**) Quantification of the proliferation marker gene’s expression (*MKI67*) relative to sporadic ALS iPSCs (n = 3 technical replicates). (**D**) Representative immunofluorescence staining of NPCs at day 6 showing expression of PAX6 (magenta) and SOX2 (green), confirming acquisition of NPC identity. Nuclei were counterstained with Hoechst (blue). Scale bar: 5 µm (n = 3 biological replicates). (**E**) Flow cytometry analysis of day-6 NPCs and iPSCs showing the expression of neural progenitor markers PAX6 and SOX2. Quantification of normalized mean fluorescence intensity (nMFI) indicates strong upregulation of both markers upon differentiation (n ≥ 5 biological replicates, mean ± SEM) (**F**) Flow cytometry analysis of day-13 NPCs showing persistence PAX6 expression and induction of the early neuronal marker TUJ1, indicating progression toward neuronal lineage commitment. Quantification of nMFI demonstrates a significant increase in TUJ1 expression (n ≥ 3 technical replicates, mean ± SEM).

We used the iPSC-derived NPC model to evaluate pridopidine’s neuroprotective potential against ER stress in ALS. The sporadic CS14isALS-Tnxx iPSC line was differentiated into NPCs using published methods [59,60]. After 6 days in culture, either Basic fibroblast growth factor (bFGF) or Epidermal growth factor (EGF) was added to ensure progenitor self-renewal and maintain NPC phenotype [61] (Figure 1A).

Immunofluorescence further demonstrated characteristic NPC morphology, including elongated cell bodies, neurite-like projections, and dense intercellular networks—features clearly distinct from the compact, colony-like organization of undifferentiated iPSCs at day 0 (Figure 1B). These molecular and morphological features collectively support a consistent neuronal progenitor phenotype.

The NPCs kept their proliferation capacity; when they reached 90% confluence, the cells were dissociated and reseeded onto new plates. qPCR analysis confirmed a sustained proliferative capacity, comparable to that observed in pluripotent cells, as indicated by *MKI67* expression (Figure 1C).

To validate that this treatment indeed preserved NPC identity, we assessed the expression of canonical progenitor markers. Flow cytometry, qPCR, and immunofluorescence confirmed sustained SOX2 and PAX6 expression, together with early neuronal marker TUJ1 (β-III tubulin), indicating retention of a stable NPC phenotype under these conditions (Figure 1D–F).

The neural induction protocol efficiently generated day-6 and day-13 NPCs (Figure 1E,F), with expression of neural markers PAX6 (52.97% ± 1.5% at day 6; 23.94% ± 0.7% at day 13), SOX2 (81.10% ± 2.3%), and TUJ1 (81.52% ± 2.6%). Together, these cytometric and morphological data demonstrate a robust and reproducible derivation of NPCs from the ALS iPSC line.

### 2.2. Pridopidine Reduces ER Stress Markers BiP and CHOP in ALS iPSC-Derived NPCs

Pridopidine was evaluated for its ability to attenuate ER stress in sporadic ALS patient-derived neural progenitor cells (ALS-NPCs). Cells were exposed to tunicamycin (1.25 µM, 16 h), and the expression of the ER stress markers BiP and CHOP was quantified by flow cytometry.

**Figure 2:**
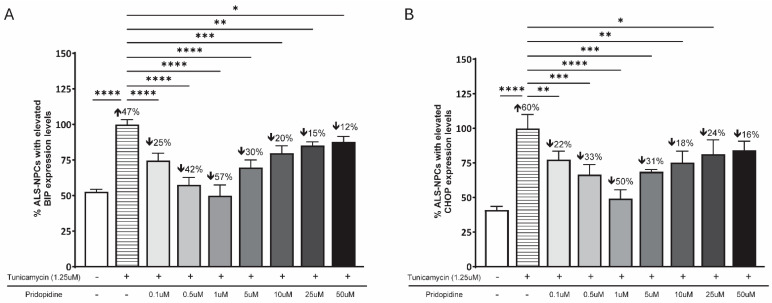
Pridopidine reduces ER stress markers BiP and CHOP in sporadic ALS-NPCs in a biphasic manner. (**A**) Percentage of ALS-NPCs with elevated BiP expression following tunicamycin treatment (1.25 µM, 16 h) and co-treatment with increasing concentrations of pridopidine (0.1–50 µM). n = 3 biological replicates. (**B**) Percentage of ALS-NPCs with elevated CHOP expression under the same conditions. n = 3 biological replicates. Data are presented as mean ± SEM.; * *p* < 0.05, ** *p* < 0.01, *** *p* < 0.001, **** *p* < 0.0001 vs. tunicamycin-treated cells (two-way ANOVA with post hoc test).

Tunicamycin exposure markedly increased the proportion of ALS-NPCs with elevated BiP expression by 47% compared with unstressed controls (*p* < 0.0001). Pridopidine significantly reduced the percentage of BiP-positive cells, with a clear biphasic dose response. The maximal effect of pridopidine was observed at 1 µM, with a 57% reduction in BiP-positive cells vs. tunicamycin-treated controls (*p* < 0.0001). Lower concentrations (0.1 and 0.5 µM) also produced significant decreases in BiP-positive cells (25%, *p* < 0.0001 and 42%, *p* < 0.000, respectively), whereas higher concentrations (5–50 µM) yielded progressively smaller effects (12–30% reduction), consistent with the characteristic biphasic response of S1R ligands (Figure 2A).

A similar pattern was observed for the effect of pridopidine on CHOP, a pro-apoptotic marker of prolonged ER stress. Tunicamycin increased CHOP-positive cells by 60% compared with unstressed controls (*p* < 0.0001). Pridopidine reduced CHOP expression in a biphasic manner, with the most pronounced effect at 1 µM pridopidine (50% reduction vs. tunicamycin-treated cells, *p* < 0.0001). Doses of 0.1 and 0.5 µM produced smaller but significant reductions (22%, *p* < 0.01 and 33% *p* < 0.001), while higher doses (5–50 µM) again showed progressively smaller effects (16–31% reduction), mirroring the S1R-dependent biphasic profile (Figure 2B).

Together, these findings demonstrate that pridopidine robustly attenuates tunicamycin-induced ER stress in ALS-NPCs, exhibiting the expected peak efficacy at 1 µM with reduced activity at lower and higher concentrations.

### 2.3. Pridopidine’s Effect on ER Stress Markers Is Mediated by Sigma-1 Receptor Activation

To determine whether the protective effects of pridopidine depend on S1R activation, ALS-NPCs were treated with tunicamycin (as before) (1.25 µM, 16 h), pridopidine (0.5 µM) and the selective S1R antagonist NE-100 (1 µM). BiP and CHOP expression levels were assessed by flow cytometry and immunocytochemistry as two complementary methodologies.

**Figure 3:**
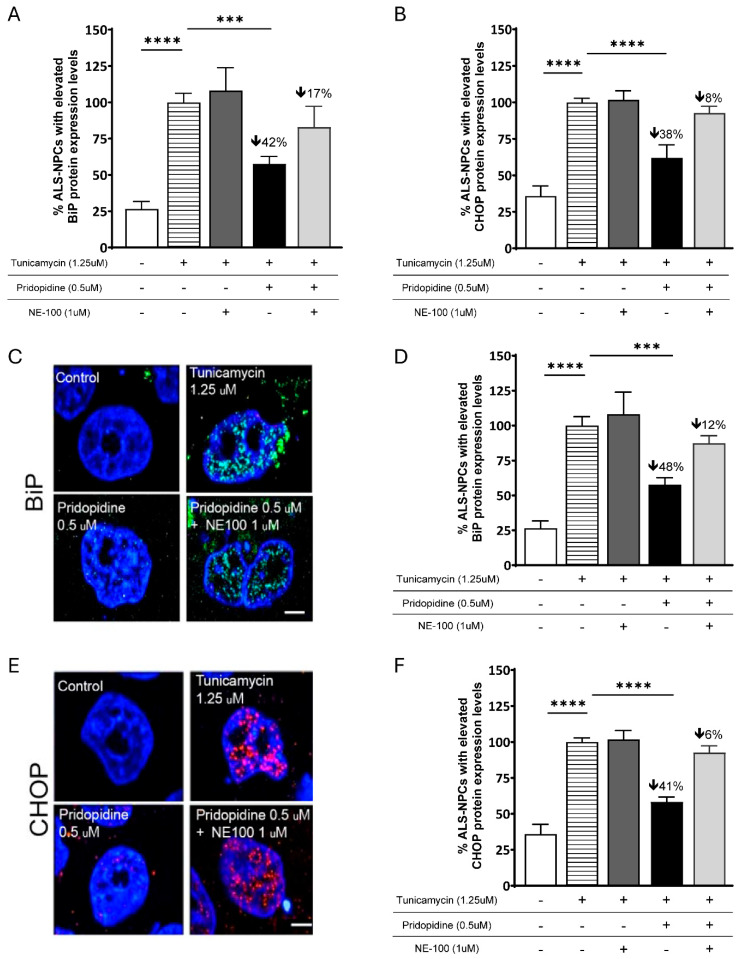
Reduction in ER stress markers BiP and CHOP by pridopidine is mediated via S1R activation. (**A**,**B**) Quantification of ER stress markers BiP (**A**) and CHOP (**B**) by flow cytometry in ALS-NPCs treated with tunicamycin (1.25 μM, 16 h) with or without pridopidine (0.5 μM) and the S1R antagonist NE-100 (1 μM). Tunicamycin markedly increased BiP and CHOP levels; pridopidine significantly reduced both markers, and NE-100 abolished this effect, indicating S1R dependence. Data represent mean ± SEM; n ≥ 5 biological replicates. Statistical analysis: **** *p* < 0.0001, *** *p* < 0.001, ns = not significant. (**C**,**E**) Representative immunofluorescence images of BiP (**C**) and CHOP (**E**). Tunicamycin induced strong BiP (green) and CHOP (red) accumulation, which was substantially reduced by pridopidine and restored by NE-100. Nuclei are counterstained with DAPI (blue). Scale bar: 5 μM. (**D**,**F**) Quantification of immunofluorescence signal for BiP (**D**) and CHOP (**F**). Pridopidine significantly decreased tunicamycin-induced BiP and CHOP intensities, whereas NE-100 attenuated this reduction. Data represent mean ± SEM; n = 3 biological replicates. Statistical analysis: **** *p* < 0.0001, *** *p* < 0.001.

Tunicamycin markedly increased BiP expression by 73% compared with unstressed controls (*p* < 0.0001) (in line with the normal variability between independent experiments (47% in Figure 2). Pridopidine significantly reduced the proportion of BiP-positive cells by 42% (*p* < 0.001) relative to stressed, untreated cultures. Importantly, co-treatment with the selective S1R antagonist NE-100 robustly attenuated this effect, resulting in a non-significant and modest 17% reduction in BiP-positive cells (Figure 3A). Immunofluorescence confirmed these findings: tunicamycin induced a strong increase in BiP staining, which was markedly reduced by pridopidine (48% reduction, *p* < 0.001), and attenuated by NE-100 administration (non-significant, 12% reduction) (Figure 3C,D).

Pridopidine showed a parallel effect on CHOP. Tunicamycin increased CHOP-positive cells by 68% compared with unstressed controls (*p* < 0.0001) (Figure 3B). Pridopidine decreased CHOP-positive cells by 38% (*p* < 0.0001), an effect antagonized by NE-100 co-treatment, yielding a non-significant 8% reduction as compared with tunicamycin alone. Consistent with the flow cytometry results, immunocytochemistry demonstrated robust CHOP induction following tunicamycin exposure, whereas it was markedly reduced by pridopidine (41% reduction, *p* < 0.001) but blocked with NE-100 (6% reduction) (Figure 3E,F).

Together, these findings from two different ER stress markers show that pridopidine’s effects are mediated via S1R signaling.

### 2.4. Pridopidine Restores Mitochondrial Membrane Potential and Reduces Mitochondrial Apoptotic Signaling in ALS-NPCs

**Figure 4:**
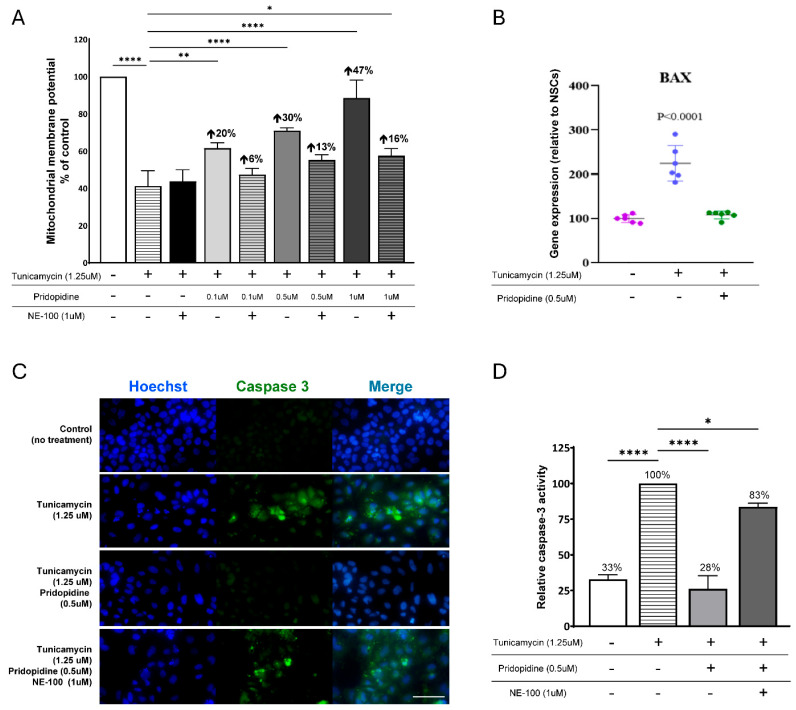
Pridopidine restores mitochondrial membrane potential and attenuates mitochondrial apoptotic signaling, including *BAX* expression and caspase-3 activation, in sporadic ALS-NPCs. (**A**) MMP was measured in ALS-NPCs following tunicamycin exposure (1.25 µM, 16 h) in the presence or absence of pridopidine (0.1–1 µM) and the S1R antagonist NE-100 (1 µM). (**B**) Relative *BAX* gene expression as measured by qPCR under the same experimental conditions as in A. Tunicamycin increased *BAX* expression; pridopidine reduced *BAX* levels, consistent with pridopidine treatment reducing mitochondrial apoptotic signaling. n = 3 biological replicates. (**C**) Representative confocal images of caspase-3 immunostaining (green) and Hoechst nuclear staining (blue) in untreated cells, tunicamycin-treated cells, and cells co-treated with tunicamycin plus pridopidine, with or without NE-100. Scale bar, 100mm. (**D**) Quantification of relative caspase-3 activity under the indicated treatment conditions. Data are expressed as % of untreated control (mean ± SEM). * *p* < 0.05, ** *p* < 0.01, **** *p* < 0.0001 vs. tunicamycin alone (one-way ANOVA with post hoc test).

Given the central role of mitochondrial dysfunction in ALS pathology, we next assessed whether pridopidine could restore mitochondrial membrane potential (MMP) in sporadic ALS-NPCs subjected to tunicamycin-induced ER stress. Tunicamycin treatment caused a profound, 58.7% reduction in MMP compared to unstressed controls (*p* < 0.0001) (Figure 4A).

Based on ER stress results, showing the strongest effect with 1 µM pridopidine, we evaluated its effect on MMP at 0.1, 0.5 and 1µM. Pridopidine significantly restored MMP levels in stressed ALS-NPCs at all three concentrations, with the strongest effect seen at 1 µM (47%, *p* < 0.0001 increase in MMP relative to tunicamycin alone). Lower concentrations (0.1 and 0.5 µM) produced more modest but significant improvements (20%, *p* < 0.01; 30%, *p* < 0.0001, respectively).

Co-treatment with the S1R antagonist NE-100 (1 μM) markedly attenuated the effect of pridopidine, but not completely abolishing it, at the optimal dose of pridopidine. Pridopidine 1 μM alone restored MMP by 47%, and when co-treated with NE-100, a weaker increase in MMP was measured at 16% (*p* < 0.05).

Importantly, the S1R antagonist NE-100 alone did not affect the TMRE signal in tunicamycin-stressed ALS-NPCs, whereas NE-100 attenuated the effect of pridopidine, supporting the notion that pridopidine’s effect is mediated via S1R.

To further assess the effect of pridopidine on mitochondrial function, we assessed the downstream mitochondrial apoptotic response to ER stress. First, we performed qPCR to assess the expression of the pro-apoptotic mitochondrial marker *BAX* (Figure 4B). *BAX* promotes mitochondrial outer membrane permeabilization, linking mitochondrial dysfunction to activation of downstream apoptotic caspases [62]. Second, we assessed caspase-3 activation, a key executioner of the apoptotic pathway (Figure 4C,D).

As shown in Figure 4B, tunicamycin exposure significantly increased BAX transcription in ALS-NPCs as compared with untreated controls (*p* < 0.0001). In contrast, pridopidine treatment significantly reduced BAX levels compared with tunicamycin-treated cells, bringing levels close to baseline (*p* < 0.0001).

Along with the increase in BAX expression, tunicamycin treatment also increased caspase-3 activity as compared with untreated controls (100% vs. 33%, *p* < 0.0001) (Figure 4C,D). Here, pridopidine treatment reduced caspase-3 activation under ER stress conditions (28%, *p* < 0.0001 vs. tunicamycin), indicating suppression of downstream apoptotic execution.

Co-treatment with the S1R antagonist, NE-100, significantly reversed the anti-apoptotic effect of pridopidine, maintaining high caspase-3 activity, further supporting pridopidine activity via S1R.

### 2.5. Pridopidine Enhances Cell Viability Under ER Stress in Sporadic ALS NPCs

To assess whether pridopidine’s effect on attenuation of ER stress and enhanced mitochondrial function translates into improved cell survival, we quantified pridopidine’s effect on the viability of ALS-NPCs exposed to tunicamycin. Cell viability was measured by flow cytometry using a fixable viability dye (FVD; amine-reactive dead cell discriminator) and normalized to unstressed controls (set at 100%).

Pridopidine at 0.1, 0.5, and 1 μM was evaluated. Tunicamycin treatment caused a profound loss of cell viability, resulting in 79% cell death (corresponding to 21% cell viability) compared with unstressed controls (*p*< 0.0001).

**Figure 5:**
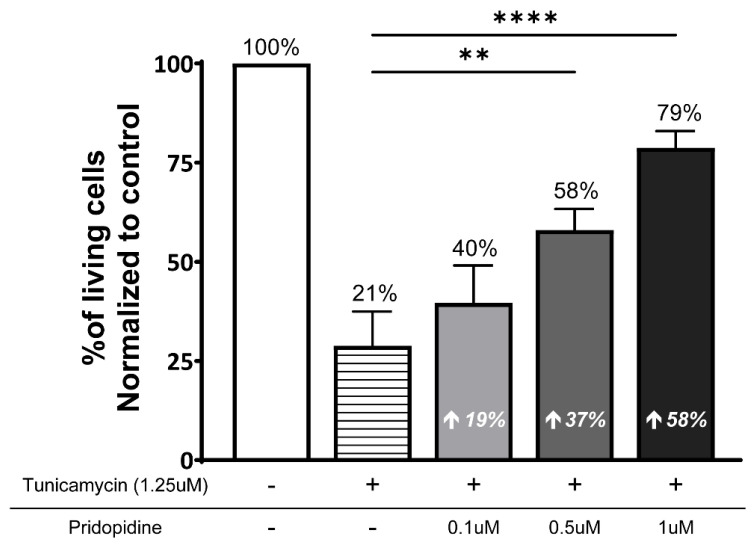
Pridopidine improves cell viability in sporadic ALS-NPCs under ER stress. Cell viability was assessed in ALS-NPCs treated with tunicamycin (1.25 µM, 16 h) with or without pridopidine (0.1–1 µM). Viability was measured using a fixable viability dye and quantified by flow cytometry, normalized to untreated controls (set to 100%). n = 3 biological replicates. Data represent mean ± SEM. ** *p* < 0.01, **** *p* < 0.0001 vs. tunicamycin alone (two-way ANOVA with post hoc test).

Pridopidine treatment provided robust, dose-dependent protection against tunicamycin-induced cell death. Consistent with prior observations on ER stress and mitochondrial membrane potential, 1 μM pridopidine produced the strongest neuroprotection, increasing ALS-NPCs’ viability by 58% relative to tunicamycin-treated cells (*p* < 0.0001). Lower concentrations exhibited more modest effects, with viability increased by 19% at 0.1 μM and 37% at 0.5 μM (*p* < 0.01) (Figure 5).

## 3. Discussion

In this study, we demonstrate that pridopidine, a highly selective and potent S1R agonist, exerts neuroprotective effects in sporadic ALS-NPCs, showing attenuation of ER stress and restoration of MMP, and overall improved cell viability. Importantly, these effects were abolished or attenuated by the selective S1R antagonist NE-100, confirming that the observed benefits of pridopidine are primarily mediated through S1R activation.

Our sporadic ALS-NPC cultures are well-characterized, expressing PAX6 and SOX2, confirming neural progenitor identity, and TUJ1 (β-III tubulin), indicating early neuronal commitment. This system represents a mixed NPC/early neuron population transitioning toward a post-mitotic neuronal state. This developmental stage is relevant for capturing early pathogenic events, including ER stress and mitochondrial dysfunction, which are known to precede overt neurodegeneration in ALS. iPSC-derived NPCs are a well-established and widely used model for studying early cellular mechanisms in neurodegenerative diseases, including ALS (e.g., Lorenz et al., 2017 [63]; Zink et al., 2021 [64]). These cells retain the patient-specific genetic background while providing a controlled and reproducible experimental system.

Consistent with reports from ALS patient tissue and from ALS nonclinical models, tunicamycin exposure markedly induced ER stress, as measured by the increased number of ALS-NPCs expressing BiP and CHOP, impaired mitochondrial function and increased cell death [11,12,13,16,17,24]. Attractive therapeutic targets in ALS focus on reducing prolonged and maladaptive ER stress, the abnormal unfolded protein response, and mitochondrial impairment. These processes contribute directly to motor neuron vulnerability and degeneration [17,18,19,65]. The ability of pridopidine to reduce ER stress and enhance mitochondrial function and cell survival is in accordance with prior published studies demonstrating a key role for S1R in regulating normal ER and mitochondrial function. These findings are aligned with the central role of S1R at MAMs, where it regulates key cellular pathways including Ca^2+^ transfer from the ER into mitochondria, ATP production, and stress adaptation [12,13,14,28,29].

We have used tunicamycin in our model, which is an accepted and widely used pharmacological agent to induce ER stress through its inhibition of N-linked glycosylation with the understanding that it does not fully recapitulate the endogenous mechanisms underlying ER stress in ALS [22]. Nevertheless, prior studies have shown that tunicamycin induction of ER stress recapitulates disease-relevant mechanisms in ALS models, supporting its use as a relevant tool for studying ER stress-associated mechanisms in patient-derived systems [18,66].

Pridopidine exhibits a biphasic dose–response profile, similar to that reported for other S1R ligands [26,50,67]. Biphasic responses have been described for S1R ligands across numerous cellular and behavioral preclinical models, including calcium signaling, neuroprotection, cognition, and antidepressant-like activity [47,50,54,56,67]. Although the underlying mechanisms are not fully resolved, this biphasic profile is considered a hallmark of S1R pharmacology and may be driven by different mechanisms including ligand-dependent receptor conformational changes, receptor oligomerization, off-target activities, membrane microdomain distribution, and different downstream signaling pathways [26,29,34]. In the present study, pridopidine exerted a maximal reduction in BiP and CHOP expression, and the largest improvement in mitochondrial membrane potential and cell viability at a dose of 1 μM, with less robust responses at lower and higher dosages of the drug [17,50,66].

Pridopidine improved mitochondrial function, as measured by restoration of MMP, and attenuated the ER-stress-induced mitochondrial apoptotic signaling pathway, as measured by reduced *BAX* expression and decreased caspase-3 activation. Importantly, pridopidine’s effects were inhibited by the selective S1R antagonist, NE-100, indicating pridopidine’s protective effects are mediated via S1R activation.

In the HEALEY ALS platform trial, while pridopidine did not show significant benefit in the primary population (including early- and late-stage ALS patients) it showed notable clinical benefits in a homogenous subgroup of faster-progressing ALS participants [68]. In the faster-progressing group, pridopidine slowed disease progression, as measured by the ALSFRS-R scale, versus the placebo group, slowed the decline in respiratory and bulbar-related functions and produced meaningful improvements in speech [68,69]. Similarly, treatment benefits were observed in HD patients. In the recent Phase 3 PROOF-HD study, pridopidine showed clinically meaningful slowing of decline across measures of progression, function, cognition and motor abilities, in a subgroup of patients who were off antidopaminergic medications [70]. Importantly, pridopidine has a favorable safety profile and clinical benefits in ALS and HD patients [37,68].

Currently approved ALS treatments remain limited. For example, Riluzole provides modest survival benefit, and tofersen, recently approved, is limited only to patients with confirmed SOD1 mutations (~2% of ALS patients). Although edaravone was approved in the US, this drug was recently withdrawn in the EU following inconclusive efficacy data [9,10]. In addition, several emerging agents such as CNM-Au8 are still in development and show mixed clinical evidence [71]. Thus, broadly neuroprotective agents remain limited. S1R represents a compelling new therapeutic target due to its critical location and function within the central nervous system (CNS). S1R is highly expressed at MAMs, where it regulates ER–mitochondria crosstalk and cellular stress responses, pathways critical for neuronal health, function, and survival, that are disrupted in ALS. Activation of S1R with selective and potent agonists such as pridopidine represents a novel approach to therapeutic intervention [12,14].

In summary, this study provides the first demonstration that pridopidine attenuates ER stress, restores mitochondrial function, and improves viability in ALS patient-derived neural progenitor cells through S1R activation. The convergence of molecular, metabolic, and neuroprotective outcomes presented here provides important mechanistic support for pridopidine’s therapeutic potential. One limitation of our study is that using a single sporadic cell line may not represent the heterogeneity of ALS. The clinical benefits observed in the HEALEY Phase 2 trial [68] further support the ongoing Phase 3 trial assessing the therapeutic potential of pridopidine in patients with ALS (PREVAiLS; NCT07322003).

## 4. Methods and Materials

### 4.1. Experimental Model and Subject Details

All experiments were performed using the human iPSC line CS14isALS-Tnxx (CSBio; W14-C146) from the Ceders-Sinai Biomanufacturing Center, Los Angeles, CA, USA, originally derived by the CS iPSC Core Repository Project (https://biomanufacturing.cedars-sinai.org/ last accessed 18 December 2025) (detailed in Table 1).

Cells were cultured under serum-free, feeder-free conditions using growth factor-reduced Matrigel-coated plates (Corning, New York, NY, USA). Cultures were maintained in mTeSR Plus medium (STEMCELL Technologies) supplemented with 1% penicillin–streptomycin (GIBCO, Miami, FL, USA). Cells were incubated at 37 °C with 5% CO_2_ and passaged every 3–4 days using gentle dissociation with recombinant trypsin EDTA solution (SARTORIUS, Beit Haemek, Israel). The medium was changed daily. Routine assessments for mycoplasma contamination were performed using a commercial diagnostic kit (LiLiF, iNtRON Biotechnology, Inc., Seongnam-si, Republic of Korea), and the pluripotency of iPSC lines was regularly confirmed via qPCR and flow cytometry analysis of pluripotent marker OCT3/4 and SOX2.

### 4.2. Differentiation of iPSCs into NPCs

The iPSCs were differentiated into NPCs using a chemically defined protocol based on [59,60], with minor modifications. Briefly, dissociated iPSCs were seeded onto Matrigel-coated plates in mTeSR plus medium and cultured until they reached approximately 80% confluence. For early NPC differentiation, cells were transitioned to a medium consisting of DMEM/F12 supplemented with 1% N2 × 100 (Thermo Fisher Scientific, Waltham, MA, USA), 1 µM Dorsomorphin (TOCRIS, Bristol, UK), 1 mM L-glutamine (GIBCO, Miami, FL, USA), 1% penicillin–streptomycin, and 0.02% heparin (STEMCELL Technologies, Cambridge, MA, USA). After 72 h, retinoic acid (RA) (100 nM, Sigma-Aldrich, Darmstadt, Germany) was added to the medium for an additional 72 h to promote late-stage NPC differentiation. During the culture period, NPCs continued to proliferate. When they reached approximately 90% confluence, the cells were dissociated and reseeded onto new Matrigel-coated plates in differentiation medium supplemented with the ROCK inhibitor Y27632 (5 μM) (Cayman Chemical Company, Ann Arbor, MI, USA) as indicated above. The medium was changed daily. After 6 days, either bFGF or EGF (1.6 nM, Thermo Fisher Scientific) was added to the medium for an additional 6 days to promote long-term NPC maintenance.

### 4.3. Quantitative Real-Time PCR (qPCR)

RNA was extracted using the PureLink RNA Mini Kit (Invitrogen) according to the manufacturer’s instructions. For cDNA preparation, the high-capacity cDNA Reverse Transcription Kit (Applied Biosystems, Waltham, MA, USA) was used according to the manufacturer’s instructions. cDNA was prepared from 0.5 to 2 mg mRNA in RNase-free conditions. RNA purity and quantity were assessed using NanoDrop (Thermo Scientific NanoDrop™) (A260/A280 1.5–2 was considered suitable for further analysis). Gene expression analysis was performed using SYBR Green gene expression assays (Sigma-Merck). Reactions were run on a StepOnePlus applied detection system (Applied Biosystems), and PCR conditions consisted of 40 cycles of 95 °C for 10 s, followed by 60 °C for 30s. The housekeeping gene *GAPDH* was used as normalization control, where relative gene expression of target genes was calculated by the delta Ct method. Primer data are detailed in Table 2.

### 4.4. Induction of ER Stress and Treatment Conditions

Day-6 NPCs at 80% confluence were washed and incubated for 6 h at 37 °C with 5% CO_2_ in fresh NPC medium containing pridopidine (Cambrex or Recipharm) at 0.1–5 μM, NE100 (Cayman Chemical Company, Ann Arbor, MI, USA) at 0.1–5 μM, or a combination of NE100 and pridopidine at 0.1–1 μM. After 6 h, tunicamycin (Cayman Chemical Company, Ann Arbor, MI, USA or Cell Signaling Technology, Boston, MA, USA) at a final concentration of 1.25 μM was added, and cells were incubated overnight at 37 °C with 5% CO_2_. Optimal tunicamycin concentrations (0.13 µM to 12.3 µM) were determined via preliminary dose–response experiments. Control samples consisted of untreated NPCs, tunicamycin-only, pridopidine-only, and NE100-only treatment conditions. Untreated and treated cells were harvested to quantify BiP/GRP78 and CHOP in flow cytometry (FACS), confocal microscopy and apoptosis assessment by qPCR analysis of *BAX* expression. Quantification of differentiated NPCs in every experiment was performed by using PAX6 and SOX2 markers by flow cytometry and/or qPCR.

### 4.5. Detection of Mitochondrial Potential

Day-6 treated and untreated NPCs, as indicated above, at 80% confluence were washed and gently dissociated. For every treatment and control, cells were counted, and 5 × 10^4^ cells were suspended per well in a 96-well plate. Following the instructions of the TMRE-Mitochondrial Membrane Potential Assay Kit (Abcam, Cambridge, UK), fluorescent dye was added to every well, and the plate was incubated at 37 °C for 30 min in the dark. Cells were washed twice in PBS and resuspended in 50 μL PBS, and fluorescence was measured using a plate reader at an excitation wavelength of 535 nm.

### 4.6. Immunostaining and Confocal Microscopy

Cells were fixed with 2% formaldehyde for 2 min and then with 4% paraformaldehyde (PFA) (Merck, Darmstadt, Germany) for 7 min, permeabilized with 0.2% (*v*/*v*) Triton-X 100 (Merck) for 5 min and blocked for 2 h in 3% BSA (Millipore, Burlington, MA, USA). Cells were then incubated overnight with primary antibodies PAX6 (Thermo Fisher Scientific; 42-6600), β-III tubulin (R&D Systems, Minneapolis, MN, USA; MAB1196), and SOX2 (GeneTex, Irvine, CA, USA; GTX627404) followed by 4 h incubation with the following secondary antibodies: donkey anti-mouse Alexa Fluor 647 and goat anti-rabbit Alexa Fluor 488 antibodies. Alexa Fluor 546 phalloidin (Thermo Fisher Scientific; A22283) was used for F-actin staining, Annexin-V (Cell Signaling Technology, 6592S) was used for detection of phosphatidylserine exposure, Hoechst was used for nuclear staining, and VECTASHIELD Mounting Medium (Vector Laboratories, Newark, CA, USA) was used for long-term preservation. Imaging was performed using a Nikon C1si laser scanning confocal microscope. For caspase-3 immunostaining, ALS-NPCs were seeded, treated and fixed as explained above. Following treatment and fixation, cells were incubated with an antibody against cleaved caspase-3 to assess apoptotic signaling. Nuclei were counterstained with Hoechst. Fluorescent images were acquired using confocal microscopy under identical acquisition settings across all treatment groups. Caspase-3 signal intensity was quantified using Fiji software (ImageJ, version 2.9.0) and expressed relative to the tunicamycin-treated condition.

### 4.7. Cell Viability and Flow Cytometry (FACS)

Cell viability was assessed using LIVE/DEAD Fixable Far Red Dead Cell Stain Kit (Thermo Fisher Scientific), which allows for the discrimination and quantification of live and dead cells in FACS. Cells were harvested, dissociated into single-cell suspensions and incubated for 30 min at 4 °C with the live/dead stain, and washed twice with FACS buffer containing PBS (Sartorius, Beit Haemek, Israel) and 10% FBS (Gibco, Miami, FL, USA). Following treatment, cells were permeabilized and fixed using the FOXP3/Transcription Staining Buffer Set (Invitrogen, Carlsbad, CA, USA). After fixation, intracellular staining was performed for ER stress marker analysis. Cells were stained with primary antibodies against BiP/GRP78 (Cell Signaling Technology; C50B12) and CHOP (Cell Signaling Technology; L63F7). Characterization of NPC differentiation included antibody staining for PAX6, SOX2 and β-III tubulin markers. Primary antibody staining was followed by incubation for 30 min with secondary antibodies: donkey anti-mouse Alexa Fluor 647 and goat anti-rabbit Alexa Fluor 488. A total of 1x10^5^ cells were incubated with Annexin-V and 7-AAD solutions at 5 mg/mL for 5–30 min. Cells were analyzed using a FACS Canto flow cytometer (BD Biosciences, San Jose, CA, USA) equipped with CellQuest Pro software, Pro (version 5.2 BD, Biosciences, San Jose, CA, USA). Fluorescence intensities were recorded for each sample, and the normalized mean fluorescence intensity (nMFI) was calculated for each experimental condition. Results were reported as the percentage of NPCs positive for the measured proteins, with comparisons made between treated and untreated cells.

### 4.8. Statistical Analysis

All experiments were performed using at least three independent biological replicates. A biological replicate is defined as an independent differentiation of iPSCs into NPCs. Technical replicates represent repeated measurements within the same experiment (e.g., replicate wells, qPCR reactions, or flow cytometry measurements).

Statistical significance between groups was determined using a two-tailed Student t-test with Bonferroni post hoc correction, or a One-Way Analysis of Variance (ANOVA) followed by Tukey’s post hoc test for multiple comparisons. Results are presented as the mean ± SEM, with *p* < 0.05 considered statistically significant. 

## Figures and Tables

**Table 1:** Summary of iPSC line.

iPSC Line Name	Gender	Age	Ethnicity	Genotype of Locus	Disease	Primary Tissue
CS14isALS-Tnxx	male	42	Unknown	Sporadic	ALS	PBMC

**Table 2:** Primer data.

Gene Name	Full Gene Name	Primer Sequence
*GAPDH*	Glyceraldehyde-3-phosphate dehydrogenase	Forward 5′-CTTTTGCGTCGCCAG-3′ Backward 5′-TTGATGGCAACAATATCCAC-3′
*MKI67*	Marker of proliferation Ki67	Forward 5′-GACAGAGGTTCCTAAGAGAG-3′ Backward 5′-AACAATCAGATTTGCTTCCG-3′
*BAX*	Bcl2 Associated X, Apoptosis Regulator	Forward 5′-AACTGGACAGTAACATGGAG-3′ Backward 5′-TTGCTGGCAAAGTAGAAAAG-3′

## Data Availability

The original contributions presented in this study are included in the article. Further inquiries can be directed to the corresponding authors.

## Abbreviations

IPSCs	induced pluripotent stem cells
NPCs	neural progenitor cells
ALS	amyotrophic lateral sclerosis
ER	endoplasmic reticulum
S1R	Sigma-1 receptor
MAM	mitochondria-associated membrane
BAX	BCL2-associated X protein
